# ASEQ: fast allele-specific studies from next-generation sequencing data

**DOI:** 10.1186/s12920-015-0084-2

**Published:** 2015-03-01

**Authors:** Alessandro Romanel, Sara Lago, Davide Prandi, Andrea Sboner, Francesca Demichelis

**Affiliations:** Centre for Integrative Biology (CIBIO), University of Trento, Trento, Italy; Department of Pathology and Laboratory Medicine, Weill Cornell Medical College, New York, USA; Institute for Computational Biomedicine, Weill Cornell Medical College, New York, USA; Institute for Precision Medicine, Weill Cornell Medical College & New York Presbyterian Hospital, New York, USA

**Keywords:** Allele-specific features, Parallel computation, Genome analysis, Transcriptome analysis, Next-generation sequencing, SNPs

## Abstract

**Background:**

Single base level information from next-generation sequencing (NGS) allows for the quantitative assessment of biological phenomena such as mosaicism or allele-specific features in healthy and diseased cells. Such studies often present with computationally challenging burdens that hinder genome-wide investigations across large datasets that are now becoming available through the 1,000 Genomes Project and The Cancer Genome Atlas (TCGA) initiatives.

**Results:**

We present ASEQ, a tool to perform gene-level allele-specific expression (ASE) analysis from paired genomic and transcriptomic NGS data without requiring paternal and maternal genome data. ASEQ offers an easy-to-use set of modes that transparently to the user takes full advantage of a built-in fast computational engine. We report its performances on a set of 20 individuals from the 1,000 Genomes Project and show its detection power on imprinted genes. Next we demonstrate high level of ASE calls concordance when comparing it to AlleleSeq and MBASED tools. Finally, using a prostate cancer dataset we report on a higher fraction of ASE genes with respect to healthy individuals and show allele-specific events nominated by ASEQ in genes that are implicated in the disease.

**Conclusions:**

ASEQ can be used to rapidly and reliably screen large NGS datasets for the identification of allele specific features. It can be integrated in any NGS pipeline and runs on computer systems with multiple CPUs, CPUs with multiple cores or across clusters of machines.

**Electronic supplementary material:**

The online version of this article (doi:10.1186/s12920-015-0084-2) contains supplementary material, which is available to authorized users.

## Background

Next-generation sequencing (NGS) provides unprecedented single base level information of the human genome and transcriptome and opens up the investigation of previously unexplored biological questions. By integrating information from individuals’ genetic makeup accessible in sequencing reads, it is possible to quantitatively estimate DNA somatic lesion clonality and infer tumor evolution, mosaicisms, or allele specific expression and binding [1-5]. Allele specific expression (ASE) is a common phenomenon observed in human cells where transcription originates predominantly from one allele [6,7]. Imprinted genes, physiological conditions (as for chromosome X inactivation) or other mechanisms affecting multiple sites of the human genome can contribute to the phenotypical human variability [6]. Specifically, ASE was demonstrated relevant to tumorigenesis in particular with respect to tumor-suppressor genes [8]. Transcript degradation by miRNA, mono-allelic disruption of a regulatory region or alternative splicing patterns, and alternative polyadenilation can initiate ASE [9-11] as well as epigenetic phenomena, like histone modifications inherited during mitosis or DNA methylation [7,12].

Available ASE analysis tools [4,5,13-15] either require trios, i.e. genomic information from individual’s parents, or solely rely on RNA-seq data with limitations in terms of exploring large datasets or in potential high false positive rates, respectively. To overcome these limitations and readily extend the analysis to large datasets, we developed ASEQ, an application that provides a complete and easy-to-use set of functionalities to optimally and rapidly perform ASE studies. We implemented an original method to identify ASE genes from paired genomic and transcriptomic NGS data that takes full advantage of a built-in fast computational engine thus reducing the effort of single base level computation, which still represents one of the major bottlenecks in NGS data analysis. Indeed, to deal with the computationally intense task of calculating reads coverage at specific chromosomal positions (namely the *pileup*), which is fundamental in ASE studies, ASEQ combines the power of multi-threaded computation with samtools C APIs, a programming library that offers rapid random access functionalities to indexed alignment files [16]. We first i) tested the performances of our tool on a selected set of 1,000 Genomes Project individuals, ii) validated its allele-specific expression detection power on imprinted genes, and iii) compared the performance with existing tools. Next, we queried paired whole exomes sequencing and transcriptomes RNA-seq data of 22 individuals to nominate ASE genes potentially involved in prostate cancer.

## Implementation

ASEQ is a command line application written in C that provides high performing NGS data retrieval features and statistical assessment of allele specific features. ASEQ includes a main execution mode, ASE, that performs the allele-specific expression computations and two auxiliary modes called PILEUP and GENOTYPE. PILEUP is the fast multi-threaded computational engine that is used by the other modes to generate pileups. The GENOTYPE mode is used to generate input information to ASE mode when necessary. PILEUP and GENOTYPE are also provided as standalone features as they proved successful in NGS pipelines that we recently applied to whole genome and to targeted sequencing data from tissue and plasma DNA [1,17].

### Parallel pileup implementation

The auxiliary mode PILEUP allows executing the pileup analysis for a list of single nucleotide positions, e.g. polymorphic positions along the genome like SNPs, using NGS data. Input and output formats (VCF, BAM, and BED) are compliant with the 1,000 Genomes Project (all specifics are outlined in the ASEQ manual and available online). Using pileup routines from samtools APIs, our application provides a built-in multi-threaded solution that optimizes the execution time when multiple CPUs or cores are available. By specifying the number of threads T to be used, the application provides two strategies for pileup computation: the *static* strategy splits the list of positions into T sub lists and initiates different threads to execute parallel pileups using a shared data structure; the *dynamic* strategy coordinates T different threads to execute parallel pileups of sequential sub lists of determined size as specified by the user using a shared data structure. While the former strategy is desirable for most scenarios, the latter one speeds-up the computation in the presence of genomic regions with high variance of completion time (e.g. regions with high levels of amplification). For each single nucleotide position in input, the PILEUP mode returns information about the read count results for each of the 4 bases A, C, G and T, the strand bias information for each base, the genomic coordinate (chromosome and position) and the unique identifier (dbsnp ID) if available. The application also provides a way to simultaneously perform multiple pileup computations on several lists of single nucleotide positions and corresponding NGS data files.

### Genotype calls

The auxiliary GENOTYPE mode determines the genotype at each input SNP position. The GENOTYPE mode is not designed to discover SNPs, but rather to compute the genotype of an input sample at known SNP positions (e.g. dbsnp catalogue). Given a list of known SNPs the application first computes the pileup from each NGS data file using the fast PILEUP computational engine and then determines the genotype calls for each sample independently. To perform genotype calls the tool offers two strategies. The first method, *htperc*, is based on alternative read counts percentages. The method calls a heterozygous genotype if the proportion of coverage of the alternative base with respect to the total coverage at that position is in the range [0.2,0.8] (default values); otherwise the method calls homozygous genotype, either for the reference or the alternative base. The second method, *binom*, implements a binomial test with probabilities p and q for the reference and the alternative allele, respectively. To account for the reference bias mapping [18], we apply default probabilities p = 0.55 and q = 0.45 (user-specific, see Additional file 1: Figure S1 and Supplementary Methods). No heterozygous genotypes are called for SNPs with reference or alternative allele coverage equal to zero. However, since this option can be too restrictive in presence of low coverage, the parameter can be set by the user, thus allowing the binomial test to be executed. Regardless of the method chosen, read counts information for reference and alternative alleles are included in the output files and are then utilized to optimize the ASE analysis. To streamline the input of the ASE mode, the GENOTYPE mode returns an output file restricted to the subset of SNPs with heterozygous genotypes. The complete list is also provided in a separate file.

### ASE analysis

**Figure 1:**
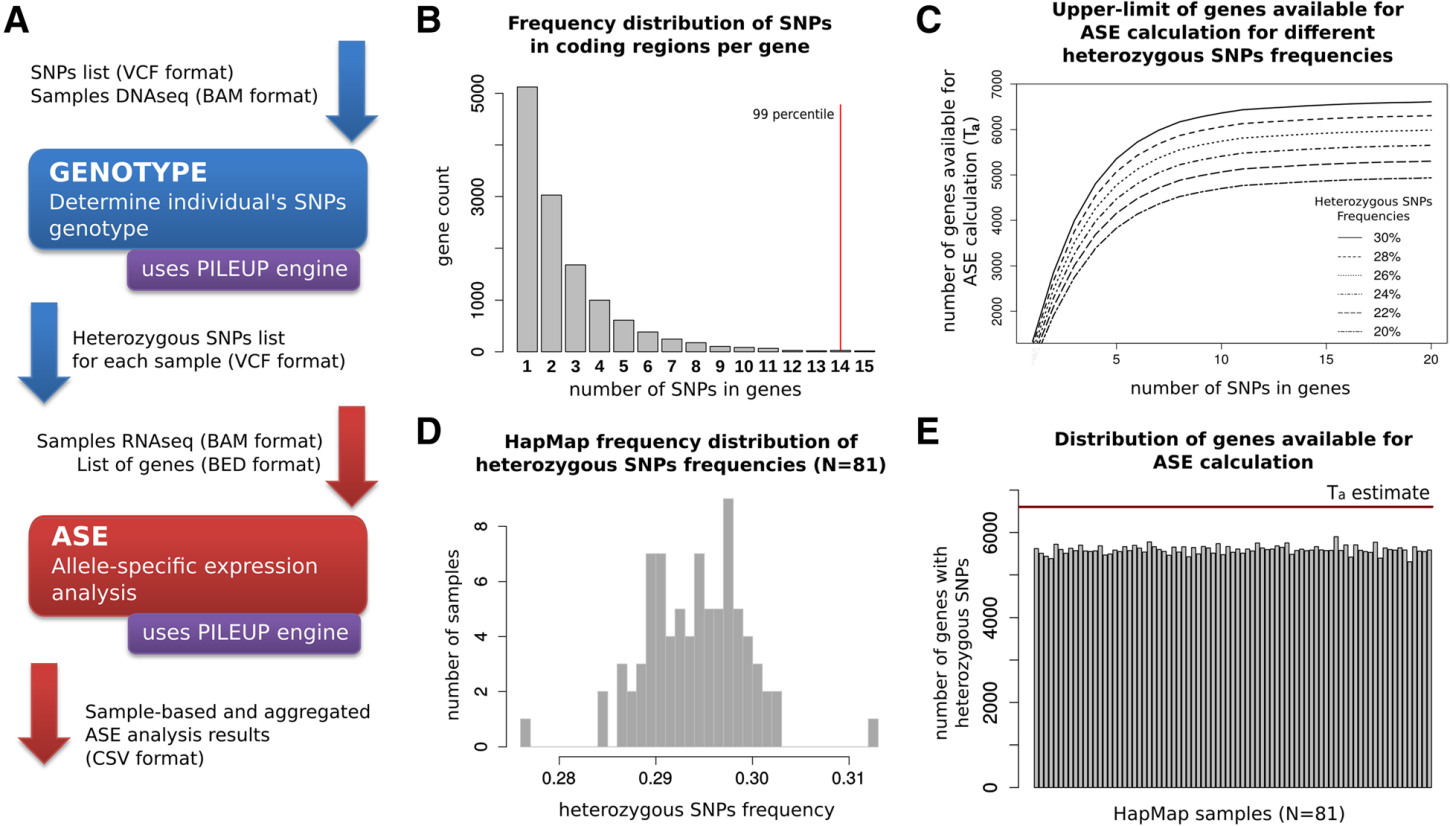
**ASEQ pipeline and detection power of SNP-based ASE studies. A)** Illustration of ASEQ pipeline used to perform ASE analysis. Given an initial list of SNPs (or genomic coordinates) and DNA-seq data, the GENOTYPE mode determines for each sample the set of heterozygous SNPs. Then, the heterozygous SNPs are analyzed with the ASE mode in the context of the corresponding matched RNA-seq samples data and a list of genes (only coding SNPs will contribute to the analysis). A final collection of sample-based and aggregated ASE results is generated. (The GENOTYPE mode works for any set of genomic positions independently from SNP annotations). **B)** Frequency distribution of coding SNPs per gene. Frequency distribution of genes containing N = 1,2,… coding SNPs based on UCSC hg19 gene catalogue and dbsnp 138 CEU. Note that the number of genes containing at most 14 SNPs corresponds to the 99 percentile of the distribution. **C)** Upper-limit of genes available for ASE calculation for different heterozygous SNPs frequencies. Upper-limit computation trends of the number of genes available for ASE calculation considering different heterozygous sites frequencies. Few SNPs per genes are enough to rapidly converge to the *T*_*a*_ estimate. **D)** HapMap frequency distribution of heterozygous SNPs frequencies. Distribution of heterozygous SNPs frequency obtained from CEU HapMap samples. **E)** Distribution of genes available for ASE calculation. Empirical distribution of ASE suitable genes is shown; horizontal line corresponds to the *T*_*a*_ for SNPs frequency equal to 30%.

The main ASE mode performs allele specific expression analysis. Two input options are implemented: (i) the *gene model* input that requires a list of coding heterozygous SNPs of the sample and a list of genes start/end coordinates; (ii) the *transcript model* input that requires a list of heterozygous SNPs of the sample and a list of transcripts with exonic coordinates. In the gene model option ASEQ matches coding SNPs and gene coordinates, whereas in the transcript model option transcript specific exon coordinates are considered for each SNP. The input list of heterozygous SNPs can be generated through the GENOTYPE mode or any other suitable SNPs genotyping tool, e.g. GATK [19]. Figure 1A shows the standard ASEQ pipeline using the gene model input.

Given a gene, the list of coding heterozygous SNPs for a study individual and the RNA-seq data file, the application performs the heterozygosity test on the RNA data at each input SNP position (using the previously described binomial method with p = q = 0.5, tunable by the user). A position is annotated as showing ASE, when a non-heterozygous call in NGS RNA-seq data is detected. To control for false positive ASE calls due to different depths of coverage between the DNA- and the RNA-seq data, the application performs an additional statistical test on the reference and alternative alleles counts proportions from the DNA and the RNA NGS data (Fisher Exact Test), whenever the DNA coverage information is available. For each sample and each gene with available heterozygous SNPs in the sample ^a^, ASEQ returns a positive ASE result if the proportion of SNPs passing the test (denoted as *ASE score*) is greater than a predefined threshold (user-specific, default equal to 0). For all gene-sample pair without available heterozygous SNPs or RNA-seq data coverage below a user-specified threshold, the application returns a flag of *not available* for ASE calculation. Additionally, when multiple samples are investigated, the application also returns an *ASE gene* flag if it shows ASE in at least N samples available for the gene ASE calculation (user-specific, default N = 1). As output, ASEQ provides both sample-based and aggregated ASE results.

For each execution mode the user can specify the minimum base quality score, the minimum read quality score and the minimum depth of coverage for the pileup computations and the significance threshold for the statistical tests used in the GENOTYPE and ASE modes (default values set to 0, 0, 1, 5%, and 5%, respectively).

## Results and discussion

### The detection power of SNP-based ASE studies

We first asked what the power of detecting ASE genes in the transcriptome of an individual through the processing of heterozygous SNPs is. First, under the assumption that one SNP per gene is sufficient to perform ASE analysis, we empirically built the distributions of ASE suitable genes on a sample basis in multiple ethnical populations from the 1000 Genomes Project and the HapMap consortium data (see Additional file 1: Figure S2 and Figure S3) and observed non-uniform behavior. Therefore, we opted for a general mathematical formulation to determine the ASE suitable genes upper bound that also models multiple SNPs per gene. Given a frequency distribution *D* of SNPs in coding regions per gene, a value *I* representing the frequency of heterozygous SNPs per individual and assuming that: (i) one SNP is sufficient to perform ASE analysis on a gene, (ii) heterozygous SNPs are uniformly distributed across the genome of an individual and (iii) SNPs are independent, we can estimate the upper-limit of the number *T*_*a*_ of genes available for ASE calculation:

$$ {T}_a={\displaystyle \sum_{i=1}^M{D}_i\ast \left(1-P\left(X=0\right)\right)}\mathrm{where}\kern0.5em X= Binom\left(i,I\right) $$

where *M* is the maximum observed number of coding SNPs overlapping a gene, *D*_*i*_ is the number of genes with *i* overlapping coding SNPs and 1 − *P*(*X* = 0) with *X* = *Binom*(*i*, *I*) is the probability that at least one of these *i* SNPs is heterozygous. To verify the validity of the formula we inspected the setting of the well represented Caucasian population in the HapMap dataset. Figure 1B shows the distribution *D* of SNPs per gene reflecting dbsnp 138 and UCSC hg19 gene catalogue and Figure 1C shows the impact of different frequencies of heterozygous SNPs on *T*_*a*_ calculation. In this setting the empirically assessed value *I* = 0.3 results in *T*_*a*_ = 6612 ASE suitable genes (23%) that is a valid over-approximation of the observed distribution (see Figure 1D, Figure 1E and Additional file 1: Supplementary Methods).

### Performances of PILEUP and GENOTYPE auxiliary methods

**Figure 2:**
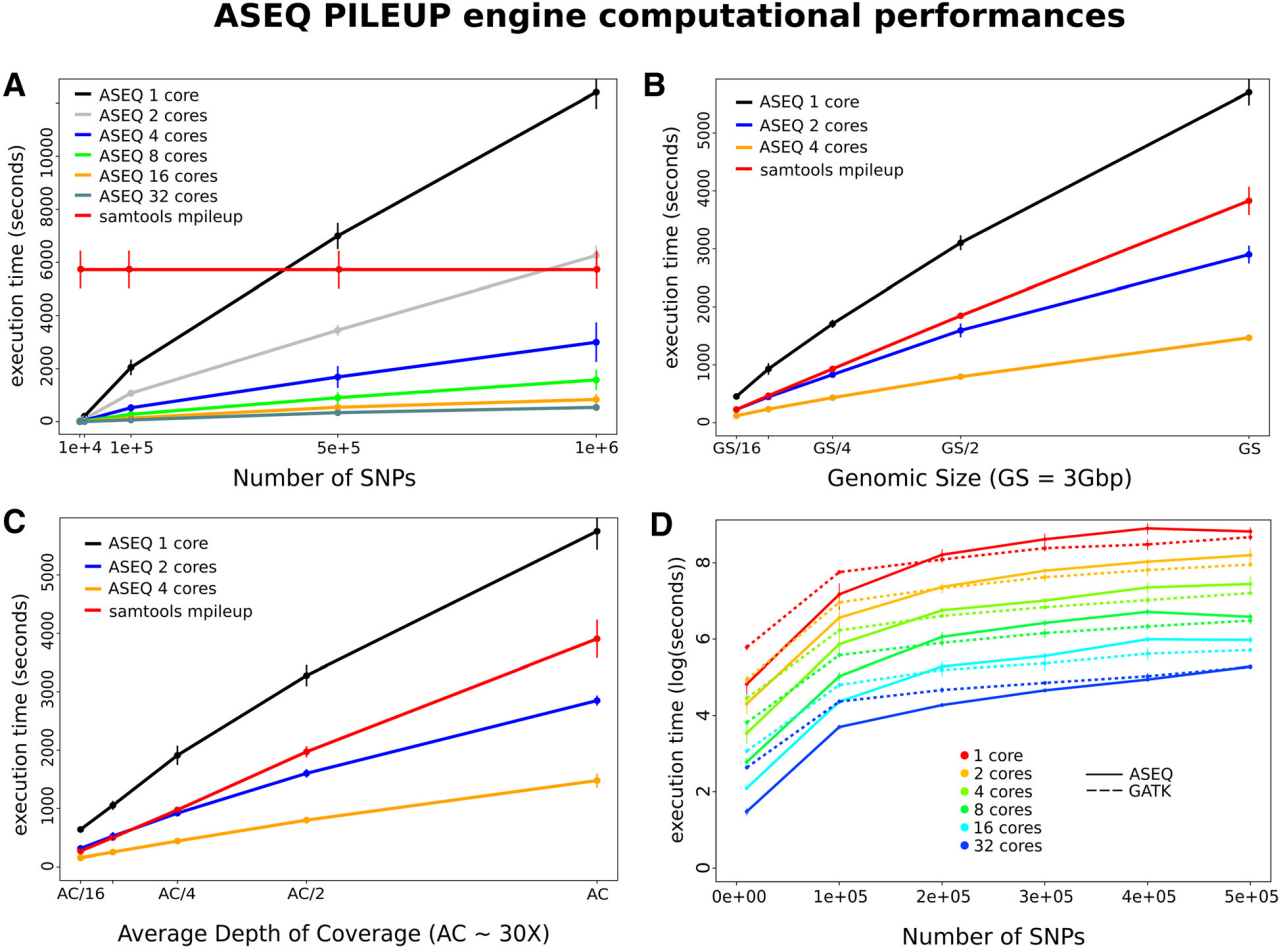
**ASEQ PILEUP engine computational performances. A)** Execution time comparison between ASEQ PILEUP mode and mpileup (Samtools, option -l to provide a SNPs list) by increasing the number of input SNPs; **B)** Execution time comparison between ASEQ PILEUP mode and mpileup (option -l was set to pass SNPs list) by increasing genomic size in Gb. **C)** Execution time comparison between ASEQ PILEUP mode and mpileup (option -l) by increasing the average depth of coverage for a human genome sample. **D)** Execution time comparison between ASEQ PILEUP mode and GATK Pileup mode.

We tested the performances of the most intensive computational task performed across all ASEQ execution modes, the multi-threaded pileup implementation PILEUP, on a multi-core machine (4 Intel® Xeon CPUs E7540 at 2.00GHz with 12 cores each in hyper-threading mode). We tested the PILEUP mode against the canonical *mpileup* samtools tool [16]. Both mpileup and PILEUP are built on top of samtools APIs. Importantly, mpileup is optimized to generate pileup of long continuous regions, whereas our approach is conceived to optimize the pileup of a list of non-contiguous single nucleotide positions. Figure 2A shows that PILEUP execution time increases linearly with the number of input SNPs, but the slope decreases logarithmically with the number of available cores. The mpileup execution time, instead is constant over different numbers of input SNPs and cores. With as few as 4 cores, PILEUP outperforms samtools when considering up to 1 million SNPs. When a single core is available, PILEUP outperforms mpileup when up to ~400,000 input SNPs are considered. On average, the number of SNPs such that PILEUP outperforms mpileup doubles by doubling the number of cores. Relevant to most single base level studies, such as ASE studies, the number of SNPs in transcriptionally active regions is within the limits where random access strategy is more effective. In addition, in the presence of multiple cores, PILEUP performances subsume mpileup ones in all the considered cases.

We next tested the performances with respect to the size of the input NGS data files adopting two strategies: random sampling of reads (Figure 2B) and random sampling of DNA coordinates (Figure 2C). The first strategy tests how PILEUP performs with NGS data files of increasing average depth of coverage, while the second tests performances with NGS data files of increasing genomic sizes. Tests were performed using 500,000 input SNPs and a human genome NGS data file (~200GB). Figures 2B and 2C show that both PILEUP and mpileup execution times increase linearly by increasing NGS data file size. In the case of PILEUP, the slope decreases with the number of available cores. Again, with multiple cores, PILEUP outperforms mpileup across all tested conditions.

For a direct comparison with other tools implementing parallel pileup computation strategies, we compared ASEQ performances against GATK Pileup module ^b^. In Figure 2D we show that for ranges of input SNPs that are reasonable for ASE studies, ASEQ execution times are comparable with GATK ones for all considered combinations of input SNPs and available cores.

**Figure 3:**
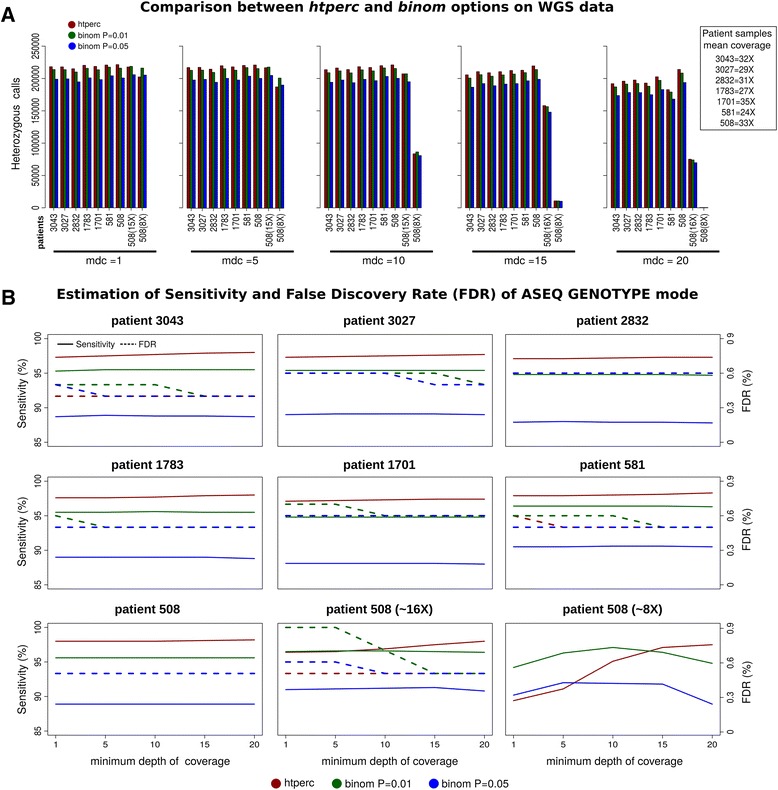
**Genotype mode performance. A)** Comparison between *htperc* and *binom* options on WGS data. Comparison of number of heterozygous calls for *htperc* and *binom* (P = 0.01 and P = 0.05) methods on 7 WGS samples (numbers identify patients IDs) from [20] increasing the minimum depth of coverage (mdc). The inset shows the samples mean coverage computed on the original BAMs on the ~2.7 million SNPs of dbsnp 138 CEU. Labels 508(16X) and 508(8X) refer to samples data where reads were computationally down-sampled with probability equal to 0.5 and 0.25, respectively, from sample 508 (original mean coverage of ~33X). **B)** Estimation of sensitivity and False Discovery Rate (FDR) of ASEQ GENOTYPE mode. Each panel shows for individual sample the sensitivity and the FDR results of ASEQ GENOTYPE mode quantified for heterozygous calls obtained on WGS data with respect to the corresponding SNP array data calls by increasing the value of minimum depth of coverage. FDR curves for sample 508 (8X) are not shown as above the maximum considered FDR across the figure panels.

To validate the performance of the GENOTYPE mode, we considered SNPs from dbsnp 138 represented on a widely used SNP array platform (see Additional file 1: Supplementary Methods). Validation was performed first on seven human prostate samples that underwent whole genome sequencing (WGS) [20] and was then extended to a larger set of 90 samples that underwent whole exome sequencing (WES) [21]. Genotype calls obtained with the two GENOTYPE methods on WGS data were compared to high quality SNP array data calls. Consistently across samples and different coverage depths, the numbers of heterozygous calls obtained by *htperc* and *binom* are comparable (Figure 3A). For each WGS sample, at depth of coverage > =10, the sensitivity of *htperc* and *binom* with stringent significance threshold remains above 95% and false discovery rate below 1% (Figure 3B). Consistently, in WES samples the mean sensitivity of *htperc* and *binom* with stringent significance threshold (P = 0.01) scored > =97% and > =92%, respectively (for depth of coverage > =10), and mean FDR scored <0.3% in both cases (Additional file 1: Figure S4). More details are available in Additional file 1: Supplementary Methods.

Overall, the tests show that our auxiliary modes are effective tools to rapidly analyze and genotype lists of known SNP loci on NGS datasets.

### ASE analysis on 1,000 Genomes Project individuals

**Figure 4:**
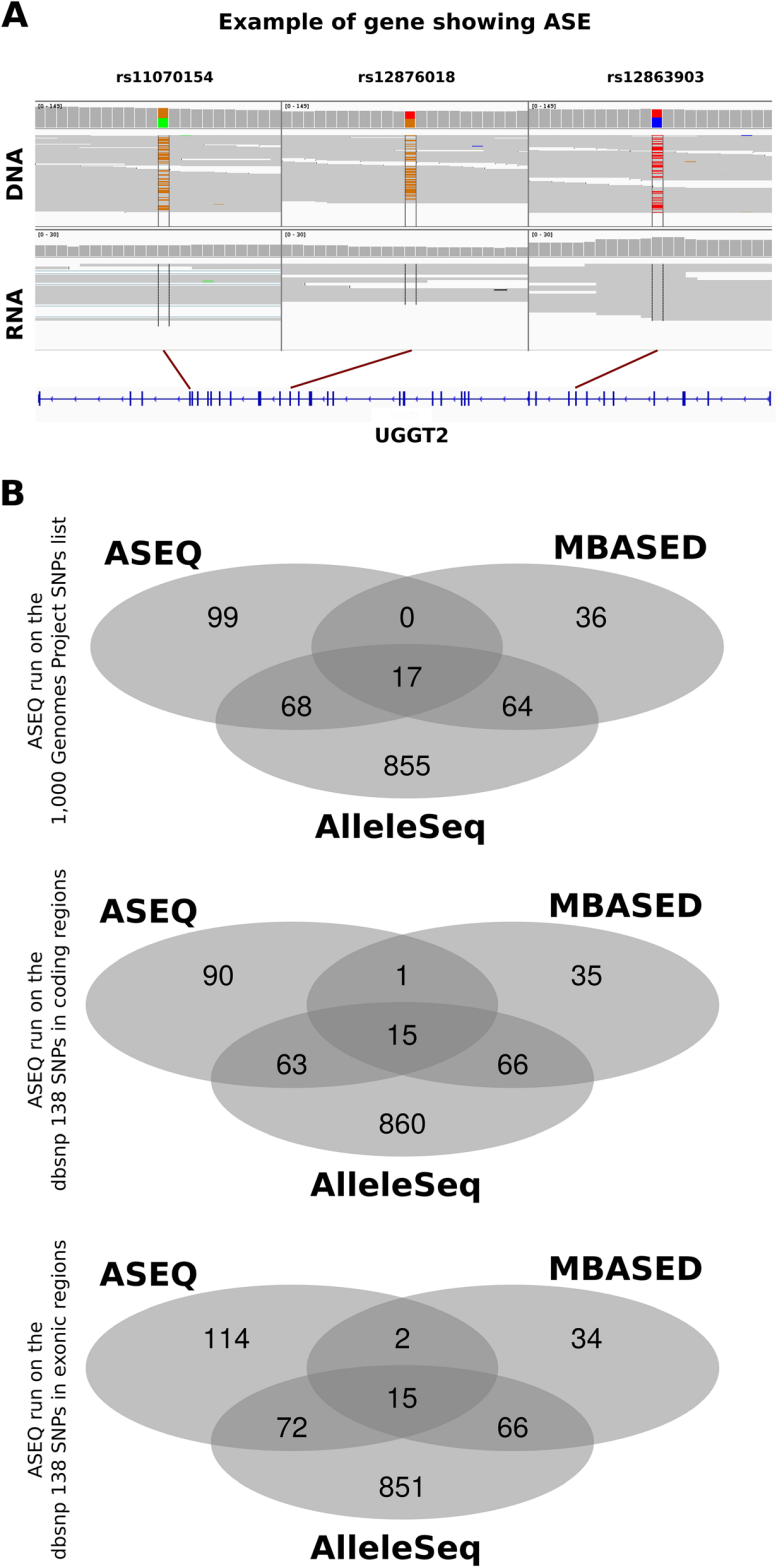
**ASE results and comparative analysis. A)** Example of gene showing ASE. We considered 1,000 Genomes Project individual NA12717 and gene UGGT2. Considering our pileup filtering parameters this gene presents three heterozygous SNPs in DNA data all showing mono-allelic transcription in RNA-seq data and is hence classified as ASE gene. **B)** Comparison with AlleleSeq and MBASED. Concordance of ASE genes detection is shown between ASEQ, AlelleSeq and MBASED. The three panels refer to ASEQ run on three different input SNPs lists. ASE genes lists for AlleleSeq and MBASED are retrieved from corresponding publications.

To investigate the extent of ASE in a human dataset, we selected 20 individuals from 1,000 Genomes Project collection for which matched WES and RNA-seq data are publicly available. We considered all coding SNPs from dbsnp 138 CEU catalogue present in UCSC hg19 gene catalogue and considered the same gene catalogue to create our gene model by means of RSEQtools [22]. Using the germline DNA-seq data, coding heterozygous SNPs were selected for each of the 20 individuals (average number across samples ~7500 SNPs, ~22% of the considered coding SNPs). Based upon RNA-seq data, genes with ASE support were identified by ASE mode (see Figure 4A for an example of identified ASE gene). Base quality > = 20, read quality > = 20, depth of coverage > = 10 and 1% of significance level for statistical tests were applied. On average (see Table 1 and Additional file 2: Table S1 for details), we detected 4.6% of genes showing ASE (ASE genes) with percentages ranging from 2.8% to 7.9%, in line with the 4.3% recently reported in [5] but lower with respect to the 19% reported in [4]. Most of the ASE genes (average 3% within range 1.8%-6.3%) show a *high ASE* score (>0.5), meaning that the majority of heterozygous SNPs on the gene support ASE. The prevalence of high ASE scores may suggest that ASE mechanisms involving most part of the whole gene (e.g. whole-gene ASE) are relatively more common.

**Table 1:** **Summary of 1**,**000 Genomes Project dataset analysis**

**Individual ID**	**DNA mean coverage**	**RNA mean coverage**	**Het SNPs**	**Available genes**	**ASE genes**	**Imprinted available genes**	**Imprinted ASE genes**	**Fisher p**-**value**
NA06994	98	46	7397	1896	94 (5%)	7	2 (28%)	0.04
NA07357	71	139	7904	1889	71 (3.8%)	9	2 (22%)	0.04
NA10847	105	104	7490	1877	101 (5.4%)	5	1 (20%)	0.2
NA11831	74	126	7604	2002	101 (5%)	3	0	-
NA11843	55	136	7361	1694	77 (4.5%)	4	1 (25%)	0.2
NA11894	148	107	6385	1683	91 (5.4%)	2	2 (100%)	0.003
NA11930	58	149	7320	1662	47 (2.8%)	7	3 (30%)	0.0008
NA11992	84	90	7724	1744	58 (3.3%)	6	0	-
NA12004	59	94	7562	1544	48 (3.1%)	3	1 (33%)	0.09
NA12043	108	77	7759	1838	91 (5%)	5	1 (20%)	0.2
NA12045	81	122	7419	1841	58 (3.2%)	3	0	-
NA12144	87	81	7844	2020	92 (4.6%)	7	2 (28%)	0.04
NA12155	83	113	7940	1802	87 (4.8%)	4	2 (50%)	0.01
NA12286	107	100	7572	1767	86 (4.9%)	8	2 (25%)	0.06
NA12348	134	124	6408	1400	50 (3.6%)	4	2 (50%)	0.008
NA12717	101	77	7467	1857	93 (5%)	4	1 (25%)	0.2
NA12750	118	89	8623	1972	108 (5.5%)	4	1 (25%)	0.2
NA12751	99	111	7396	2115	97 (4.6%)	5	3 (60%)	0.001
NA12842	182	141	7728	2074	163 (7.9%)	7	2 (28%)	0.1
NA12874	69	129	7297	1738	85 (4.9%)	4	1 (25%)	0.2
**Average**	**96.05**	**107.75**	**7510**	**1820.75**	**84.9** **(4.6%)**	**5.05**	**1.45** **(29%)**	-

For each individual we report the mean coverage in WES and RNA-seq data computed at SNP positions and the number of heterozygous SNPs identified from WES. Then we report the number of genes found available for ASE calculation along with the number of genes identified as ASE genes with corresponding percentages. Finally, we report the number of imprinted genes we found available for ASE calculation, the number of these that are identified as ASE genes and the p-values obtained by testing the significance of proportion of imprinted ASE genes with respect to the overall ASE genes proportion (no test is performed when 0 imprinted ASE genes are detected).

In the absence of a *gold*-*standard* to test ASE analysis tools, we quantify ASEQ performances by first comparing it with a trio analysis based tool, AlleleSeq [4], and with a RNA-seq data only tool, MBASED [5], and then by measuring its power in detecting imprinted genes. To explore the comparison with AlleleSeq and MBASED we focused on the 1,000 Genomes Project individual NA12878. ASE genes lists for AlleleSeq and MBASED were retrieved from corresponding studies while for ASEQ we considered germline WES data available from the 1,000 Genomes Project collection and RNA-seq available from Rozowsky et al. study (see Additional file 1: Supplementary Methods). ASEQ pipeline processed WES data with the GENOTYPE mode on three different input SNPs lists (1,000 Genomes Project SNPs list, dbsnp 138 SNPs in coding regions and dbsnp 138 SNPs on exonic regions). We obtained ASE percentages in the range 6%-7.1% (statistical significance level threshold at 1%; see Tables 2, S2, S3 and S4 for details), in line with what reported in [5]. Figure 4B illustrates the distributions of potential ASE genes as revealed by ASEQ, MBASED and AlleleSeq. Overall 17 ASE genes were commonly detected by all three methods. When restricting the analysis on ASEQ and MBASED common genes (i.e., genes for which both methods provide an ASE call ^c^, see Additional file 1: Supplementary Methods for details), ASEQ detects ~60% of MBASED detected genes with an intersection percentage of 24% (enriched with respect to the baseline ASEQ detection percentage, *P* < 10^− 8^ Fisher Exact Test), supporting a significant concordance between the two methods (see Additional file 1: Supplementary methods for details). For ASEQ versus AlleleSeq comparison, we implemented a different strategy based on resampling statistical method (see Additional file 1: Supplementary Methods for details) as the initial gene list from [4] is not available. An intersection percentage of ~46% (*P* < 10^− 4^) further supports the ASE detection power of ASEQ.

**Table 2:** **Summary of NA12878 individual analysis**

**Input SNP list**	**DNA mean coverage**	**RNA mean coverage**	**Het SNPs**	**Available genes**	**ASE genes**	**Available imprinted genes**	**Imprinted ASE genes**
1,000 Genomes Project	88	39	16016	3071	184 (6%)	10	1
Coding dbsnp 138	100	41	7465	2403	169 (7%)	9	2
Exon dbsnp138	90	40	9372	2840	203 (7.1%)	13	4

We report ASE analysis results on NA12878 individual for all combinations of input SNP list considered. For each combination we report the mean coverage in WES and RNA-seq data computed at SNP positions and the number of heterozygous SNPs identified from WES. Then we report the number of genes found available for ASE calculation along with the number of genes identified as ASE genes with corresponding percentages. We also report the number of imprinted genes we found available for ASE calculation and the number of these that are identified as ASE genes.

Finally we investigated to what extent ASEQ is able to detect known imprinted genes, using the genomic imprinting website (geneimprint.com). On average (see Table 1 for details) 30% (average 5, range from 2 to 9) of the genes available for this analysis were detected by ASEQ. Considering all samples where at least one imprinted gene was detected, the average detection proportion is 8 times higher than the baseline ASEQ detection; despite the small number of imprinted genes, the difference in the proportions is statistically significant for half of the individuals (*P* < 0.05 Fisher Exact Test, see Table 1 for details). For the individual NA12878, MBASED detects 3 out of 8 imprinted genes, while AlleleSeq identifies 5 imprinted genes. In both cases ASE detection proportions are in line with ASEQ results (see Table 2).

Altogether, we assessed that ASEQ detection performance are largely satisfactory and that running time is advantageous for large scale ASE analysis (computation of the 20 individuals from the 1,000 Genomes Project using 20 cores ran in less than 25 minutes).

### ASE analysis on a prostate cancer dataset

**Figure 5:**
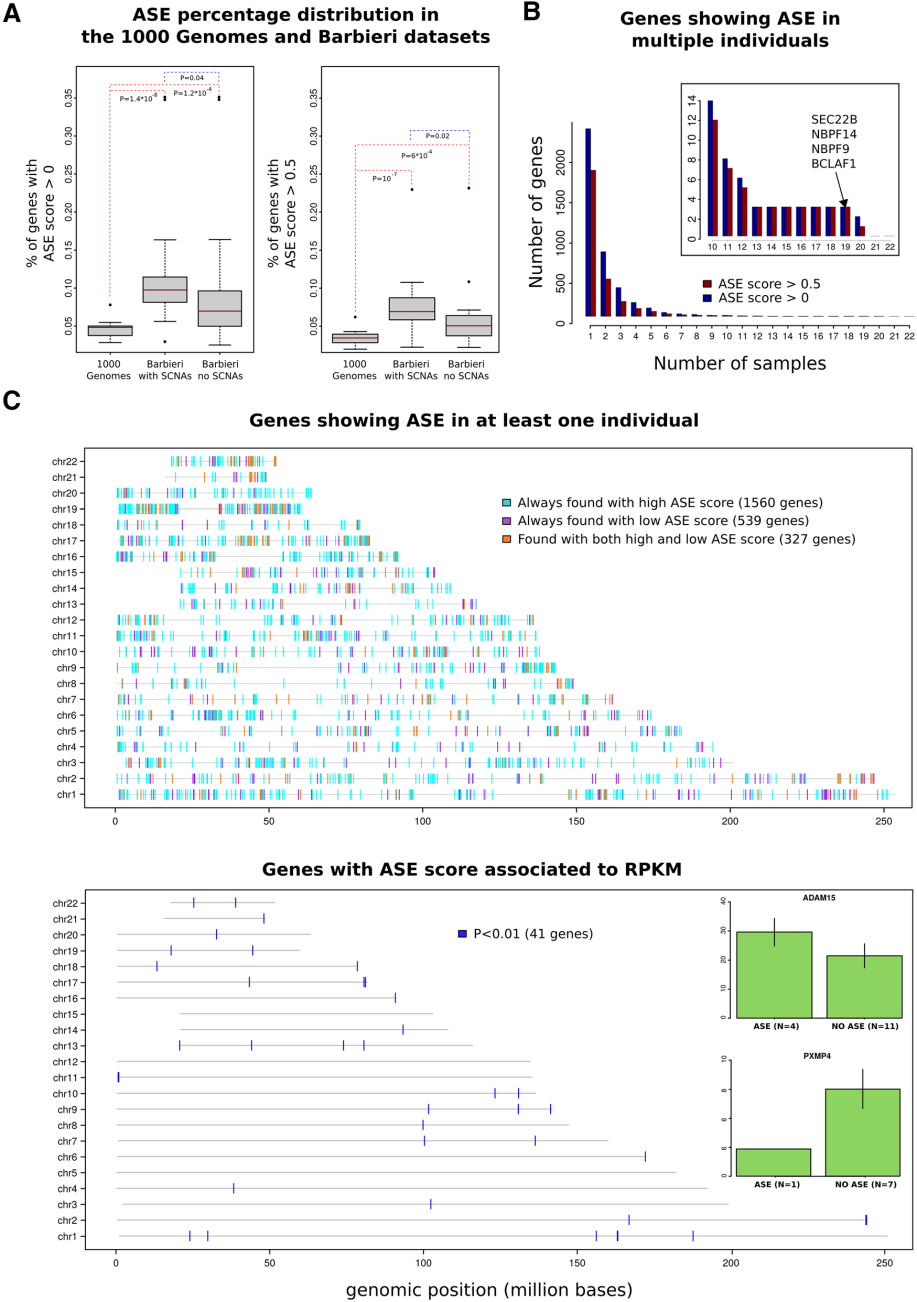
**Case study assessment of ASE genes. A)** ASE percentage distribution in 1,000 Genomes Project and Barbieri et al. dataset. Difference in ASE percentage distribution among samples in the 1,000 Genomes Project dataset, the Barbieri et al. dataset and the Barbieri et al. dataset with SCNAs filtered out. Comparison is made both for overall ASE genes and high score ASE genes. Wilcoxon statistical test is used to compare the distributions. **B)** Genes showing ASE in multiple individuals. Frequency distribution of genes showing ASE in at least N (1,2,..,22) individuals, divided by ASE genes and ASE genes with high score. The inset highlights the tail of the distribution and lists the genes that show ASE in at least 13 to 19 individuals. **C)** Genes showing ASE in at least one individual (top). Genes with ASE score associated to RPKM levels (bottom). Top panel shows the genomic localization across the human genome of all the ASE genes with low score and ASE genes with high score. Bottom panel shows the distribution across the genome of all the ASE genes with score associated to the corresponding RPKM transcript level (P < 0.01). The inset shows two prostate cancer related ASE genes with corresponding RPKM transcript levels differences.

To explore the extent of ASE in a tumor dataset, we queried matched germline WES and tumoral RNA-seq data for 22 prostate cancer patients from the Barbieri et al. study [21]. As previously, we considered all coding SNPs from dbsnp 138 CEU catalogue present in UCSC hg19 gene catalogue and considered the same gene catalogue to create our gene model by means of RSEQtools [22]. Using the germline DNA-seq data, coding heterozygous SNPs were selected for each of the 22 individuals (average number across samples ~7600 SNPs, ~23% of the considered coding SNPs). Base quality > = 20, read quality > = 20, depth of coverage > = 10 and 1% of significance threshold for statistical tests were applied. On average (see Table 3 and Additional file 2: Table S5 for details), we detected 11.6% of genes ASE genes with percentages ranging from 3% to 35%. Also in this case most of the ASE genes (average 8% within range 2%-24%) show a *high ASE* score (>0.5). As the distribution of ASE genes percentages in the Barbieri et al. dataset was significantly higher than in the 1,000 Genomes Project dataset (Figure 5A) and the sequencing characteristics comparable (see Table 1 and Table 3), we wondered to what extent the presence of somatic copy number aberrations (SCNAs) could have affected the analysis; for instance, a gene harboring a monoallelic deletion would appear as an ASE gene. We considered SCNAs profiles reported in the original study [21] and filtered out genes with genomic coordinates overlapping aberrant segments (copy neutral loss of heterozygosity LOH are not considered as they are infrequent in localized prostate cancer). Interestingly, we still detected 9.8% of ASE genes on average with percentages ranging from 3% to 34%. Again, most of the ASE genes (average 6.8% within range 2%-24%) show a *high ASE* score (>0.5) (see Additional file 2 Table S6 for details). Although lower, the distribution of ASE genes in the filtered Barbieri et al. dataset still is significantly higher than in the 1,000 Genomes Project dataset (Figure 5A). Overall these results are in accordance with what reported in [5].

**Table 3:** **Summary of Barbieri et al. dataset analysis**

**Individual ID**	**DNA mean coverage**	**RNA mean coverage**	**Het SNPs**	**Genes Available**	**ASE Genes**
01-28R	109	107	7665	2138	259 (12.1%)
03-1426R	103	126	7677	2214	180 (8.1%)
03-2345R	93	124	7719	2594	78 (3%)
04-1084 L	102	115	7744	2610	275 (10.5%)
04-1243 L	103	108	7590	2399	151 (6.3%)
05-3595TTZ	165	114	7685	2499	146 (5.8%)
05-3852 L	104	114	7554	2169	223 (10.3%)
06-1749TR	167	113	7560	2688	258 (9.6%)
07-144R	107	99	7527	2540	322 (12.7%)
07-360TZ	108	139	7581	2364	160 (6.8%)
07-837 L	87	115	7506	2278	208 (9.1%)
2661_Dt	185	82	7829	2162	214 (9.9%)
2682_A	183	86	7701	1580	181 (11.5%)
2740_A	174	84	7572	1871	105 (5.6%)
2761_D	189	83	7552	2180	184 (8.4%)
2858_C	181	68	8084	2017	330 (16.4%)
2872_D	142	69	7663	1516	137 (9%)
2916_At	176	69	7722	1860	647 (34.8%)
3023_B62	193	96	7616	1956	688 (35.2%)
3026_B56	172	79	7851	1787	160 (9%)
3035_B53	163	69	7582	1679	177 (10.5%)
3036_B51	179	86	7618	1793	187 (10.4%)
**Average**	**142.15**	**99.5**	**7669.9**	**2171.1**	**239.5** **(11.6%)**

For each individual we report the mean coverage in WES and RNA-seq data computed at SNP positions and the number of heterozygous SNPs identified from WES. Then we report the number of genes found available for ASE calculation along with the number of genes identified as ASE genes with corresponding percentages.

While ~45% of the genes shows ASE in at least 2 individuals, only ~0.5% are detected in at least half of the individuals (Figure 5B) including members of the Neuroblastoma breakpoint family (NBPF9 and NBPF14) that are deregulated in several cancer types [23].

We next asked if individuals with evidence of ASE for a specific gene demonstrate corresponding differential transcript levels (Figure 5C, see Additional file 1: Supplementary Methods). The top ranked associations (*P* < 0.01) included two genes implicated in prostate cancer; specifically increased *ADAM15* and decreased *PXMP4* expressions [24-27] (Figure 5C bottom panel inset). The metalloproteinase *ADAM15* mRNA and protein levels are over-expressed in prostate cancer and its expression is significantly increased during metastatic progression. *PXMP4* is a peroxisomal membrane protein that undergoes hypermethylation associated with gene silencing during cancer progression. Overall, these findings support the hypothesis that ASE is enriched in cancer cells.

## Conclusions

We presented a tool to rapidly screen NGS datasets for allele specific expression studies. This tool can also be applied to investigate eQTL [28]. Systematic assessment of ASEQ performance showed the efficacy and reliability of the approach on multiple datasets and identified potential cancer related ASE genes. The tool can be used within any NGS pipeline that runs on computer systems with multiple CPUs, CPUs with multiple cores, or across clusters of machines. As future work we will apply ASEQ to identify tissue and cancer specific ASE genes and explore its efficacy in detecting allele-specific binding (ASB) patterns in cancer.

### Availability and requirements

**Project name:** ASEQ

**Project home page:**http://demichelislab.unitn.it/ASEQ

**Operating system(s):** Platform independent

**Programming language:** C

**License:** MIT

### Endnotes

^a^Note that a gene may span multiple SNPs.

^b^Note that while ASEQ PILEUP mode returns the read count for each base separately, to have the same output data GATK Pileup mode would require an additional processing step that for simplicity here is not considered in the overall GATK Pileup execution time.

^c^Different tools embed different preprocessing, filtering and processing pipelines along with different set of conditions to be satisfied for an ASE call to be made. This may result in different set of analyzable genes.

## Additional files

Additional file 1: Figure S1. Distribution of mean reference allelic fraction from 111 normal WES samples of Barbieri et al. dataset. **Figure S2:** Distribution of number of genes containing at least one heterozygous coding SNP across different populations from 1000 Genomes Project data. Genotyping data of ~600000 coding SNPs for 848 samples across 9 populations were considered. For each sample the number of genes containing at least one heterozygous SNP is computed using the UCSC hg19 genes catalogue as reference. **Figure S3:** Distribution of number of genes containing at least one heterozygous coding SNP across different populations from HapMap data. Genotyping data of ~200,000 coding SNPs for 736 samples across 9 populations were considered. For each sample the number of genes containing at least one heterozygous SNP is computed using the UCSC hg19 genes catalogue as reference. Figure S4: Sensitivity and FDR of GENOTYPE ASEQ method calculated on 90 WES sample from Barbieri et al. dataset. Additional file 2: Table S1. ASEQ ASE analysis results for the 1,000 Genomes Project dataset. **Table S2:** ASEQ ASE analysis results for the NA12878 individual (1,000 Genomes Project SNPs input list). **Table S3:** ASEQ ASE analysis results for the NA12878 individual (dbsnp coding SNPs input list). **Table S4:** ASEQ ASE analysis results for the NA12878 individual (dbsnp exonic SNPs input list). **Table S5:** ASEQ ASE analysis results for the Barbieri et. al dataset. **Table S6:** ASEQ ASE analysis results for the Barbieri et. al dataset without genes in somatic copy number alterations.

## Abbreviations

NGS: Next-generation sequencing
ASE: Allele-specific expression
WGS: Whole genome sequencing
WES: Whole exome sequencing
UCSC hg19 gene catalogue: UCSC hg19 knownGenes catalogue
