# Supplementary material for: ASEQ: fast allele-specific studies from next-generation sequencing data
Source: BMC Med Genomics. 2015 Mar 1;8:9. doi: 10.1186/s12920-015-0084-2 (PMC4363342; doi:10.1186/s12920-015-0084-2)
Supplement: Additional file 1: Figure S1. — Distribution of mean reference allelic fraction from 111 normal WES samples of Barbieri et al. dataset. Figure S2: Distribution of number of genes containing at least one heterozygous coding SNP across different populations from 1000 Genomes Project data. Genotyping data of ~600000 coding SNPs for 848 samples across 9 populations were considered. For each sample the number of genes containing at least one heterozygous SNP is computed using the UCSC hg19 genes catalogue as reference. Figure S3: Distribution of number of genes containing at least one heterozygous coding SNP across different populations from HapMap data. Genotyping data of ~200,000 coding SNPs for 736 samples across 9 populations were considered. For each sample the number of genes containing at least one heterozygous SNP is computed using the UCSC hg19 genes catalogue as reference. Figure S4: Sensitivity and FDR of GENOTYPE ASEQ method calculated on 90 WES sample from Barbieri et al. dataset. [file 12920_2015_84_MOESM1_ESM.docx]

Supplementary Methods

**ASEQ: fast allele-specific studies**

**from next-generation sequencing data**

Alessandro Romanel^1^, Sara Lago^1^, Davide Prandi^1^, Andrea Sboner^2,3,4^ and Francesca Demichelis^1,3,4,*^

^1^Centre for Integrative Biology (CIBIO), University of Trento, Italy

^2^Department of Pathology and Laboratory Medicine, Weill Cornell Medical College, New York, USA

^3^Institute for Computational Biomedicine, Weill Cornell Medical College, New York, USA

^4^Institute for Precision Medicine, Weill Cornell Medical College & New York Presbyterian Hospital, New York, USA

**Read mapping bias estimation**

Reference mapping bias was estimated considering 111 normal samples that underwent whole exome sequencing (WES) [1]. Using PILEUP mode we considered ~2.7M SNPs from dbsnp 138 CEU catalogue and computed for all samples the mean reference allelic fraction:

$$\frac{ref}{ref+alt}$$

across all positions showing a minimum depth of coverage of 20 and reference allelic fraction >=20% and <=80%. Based on the distribution depicted in Figure S1 we set the reference genome mapping bias to 0.55.

**CEU HapMap samples processing**

We downloaded from HapMap website the set of genotyping data (Release 28) for CEU population (N=171). We extracted only the samples presenting an overall SNP call rate >90%, resulting with 81 samples. We then focused only on dbsnp 138 CEU catalogue SNPs and selected only the SNPs presenting an overall call rate >90%, resulting with a set of ~2.6M SNPs.

For each sample we computed:

- The frequency of heterozygous SNPs as the ratio of the number of heterozygous calls and the number of total calls;
- The number of genes containing at least one heterozygous SNP using the UCSC hg19 gene catalogue as reference.

**GENOTYPE mode validation**

To validate the performance of the GENOTYPE mode, we considered SNPs from dbsnp 138 CEU represented on the genotype Affymetrix platform and seven human prostate samples that underwent whole genome sequencing (WGS) [2] for which array data are also available. Genotype calls obtained with the two GENOTYPE methods on WGS data were compared to high quality SNP array data calls. We considered data from Affymetrix SNP 6.0 platform which provides ~900,000 SNP, of which ~800,000 are annotated in dbsnp 138 CEU catalogue. SNP genotype calls from SNP array data was performed using the standard pipeline provided by APT tools. Manufacturer claims a call rate of 99.8% for the platform genotype calls; this percentage that has been also confirmed in [3]. Moreover, several studies have been successfully performed using the Affymetrix platform [4,5] by our group. Hence this makes us confident that the Affymetrix SNP genotype calls can be used as a high quality reference dataset to compare our ASEQ genotype calls with.

Figure 3A compares the number of heterozygous calls obtained by *htperc* and *binom* methods (with two different significance thresholds) across all samples by increasing the minimum depth of coverage (mdc) considered in the pileup computation (standard quality filters of 20 for base and read quality were applied). Consistently across samples and different coverage depths, the numbers of heterozygous calls are comparable. As expected, a significance threshold of 5% for *binom* test results in less heterozygous calls with respect to a threshold at 1%. Indeed, being the null hypothesis that the two alleles are heterozygous, a higher threshold will provide more sensitivity in rejecting it, hence supporting the alternative hypothesis that the two alleles are not heterozygous.

To assess the genotype performances with respect to SNP array data, we measured both *sensitivity* (percentage of heterozygous SNP array calls that are called heterozygous by ASEQ) and *false discovery rate (FDR)* (percentage of heterozygous calls by ASEQ that are not called heterozygous by SNP array data). Figure 3B shows that for each sample at depth of coverage >=10, the sensitivity of *htperc* and *binom* with stringent significance threshold remains above 95% and FDR below 1%. By increasing the minimum depth of coverage we have less heterozygous calls overall. In addition, when the mean sample depth of coverage is low (8-16X) the *binom* method tends to be more precise than *htperc*.

Further, we extended the validation to a larger set of 90 samples that underwent whole exome sequencing (WES) [1], by considering ~7,000 coding SNPs of dbsnp 138 CEU available on the Affymetrix platform. Consistently with the WGS based analysis, mean sensitivity of *htperc* and *binom* with stringent significance threshold (P=0.01) scored >=97% and >=92%, respectively (for depth of coverage >=10), and mean FDR scored <0.3% in both cases (Figure S4). Noteworthy, using 30 cores of the in-house multi-core machine, genotyping of the ~7,000 coding SNPs on 90 samples with 3 different methods and 5 minimum depth of coverage values (for a total of 1350 genotyping runs) was computed in less than 6 hours.

**RNA-seq data processing**

RNA-seq FASTA files where downloaded from *http://archive.gersteinlab.org/proj/AlleleSeq/RNAseq/*. Alignment was performed using TopHat v2.0.10 [6] and duplicated reads were removed using Picard v1.92(1464) (*http://broadinstitute.github.io/picard/*). Samtools [7] were used to index the resulting BAM file.

**MBASED comparison**

MBASED analysis of sample NA12878 [8] reports a list of 2560 genes for which an ASE call is available. Considering the union of phased ASE genes (N=110) and unphased ASE genes (N=115) they provide overall a list of 117 ASE genes. The analysis we performed with ASEQ on sample NA12878 instead reports: a list of 2886 genes for which an ASE call is available with 184 ASE genes if the 1,000 Genomes Project SNPs list is considered; a list of 2274 genes for which an ASE call is available with 169 ASE genes if the dbsnp 138 SNPs in coding regions is considered; a list of 2662 genes for which an ASE call is available with 203 ASE genes if the dbsnp 138 SNPs in exonic regions is considered.

Considering the 1,000 Genome Project input SNPs list (similar trends are observed using the other two input SNPs lists) and focusing the analysis on common genes, i.e. the genes for which both methods provide an ASE call, we observed that ASEQ and MBASED have 1473 common genes of which 72 show allele specific expression in ASEQ and 28 in MBASED. Considering that the two methods detect 17 common ASE genes, it results that ASEQ detects 60% (17/28) of MBASED detected genes with an ASEQ intersection percentage of 24% (17/72) that results enriched with respect to the baseline ASEQ detection percentage ($P<10^{-8}$, Fisher Exact Test).

**AlleleSeq comparison**

To test whether the intersection of ASEQ and AlleleSeq ASE genes lists is statistically significant we implemented a resampling statistical test. Given N to be the total genes ASEQ finds as available for ASE calculation, N’ to be the subset of ASEQ ASE genes and M the number of those that are in common with AlleleSeq, we generated 10,000 lists containing N’ random genes selected among the N ones. We then generated a reference distribution D by intersecting each random list with AlleleSeq ASE genes list and computed statistical significance of ASEQ ASE genes list intersection with the formula:

$$P=\frac{|\{i|i\in D\wedge i>=M\}|}{|D|}$$

We observed that all three ASE genes lists we obtained running ASEQ ASE analysis on the three different input SNPs list (1,000 Genome Project SNPs, dbsnp 138 SNPs in coding regions and dbsnp 138 in exonic regions) have statistically significant intersections with AlleleSeq ASE genes, with $P<10^{-4}$ in all three cases.

**RPKM calculation and analysis**

RPKM levels were computed with RSEQtools [9] using the standard RSEQtools pipeline and considering the UCSC hg19 gene model. Association analysis between ASE gene status across individuals and related gene RPKM levels was computed using a statistical test that combines a Mann-Whitney test, an allelic test, a dosage test and a Pearson correlation test. We identified a total of 41 genes with P<0.01 (at least one test is significant) which, considering the low number of samples available in the analysis, we ranked as top associated genes. In the analysis we considered only genes that show ASE in at least one individual and do not show ASE in at least one individual. Moreover we excluded all the genes that have RPKM value less than 1 in all individuals.

**References**

1. Barbieri CE, Baca SC, Lawrence MS, Demichelis F, Blattner M, Theurillat J-P, et al. Exome sequencing identifies recurrent SPOP, FOXA1 and MED12 mutations in prostate cancer. Nature genetics. 2012;44:685–9.

2. Berger MF, Lawrence MS, Demichelis F, Drier Y, Cibulskis K, Sivachenko AY, et al. The genomic complexity of primary human prostate cancer. Nature. 2011;470:214–20.

3. Nishida N, Koike A, Tajima A, Ogasawara Y, Ishibashi Y, Uehara Y, et al. Evaluating the performance of Affymetrix SNP Array 6.0 platform with 400 Japanese individuals. BMC genomics. 2008;9:431.

4. Setlur SR, Chen CX, Hossain RR, Ha JS, Van Doren VE, Stenzel B, et al. Genetic variation of genes involved in dihydrotestosterone metabolism and the risk of prostate cancer. Cancer Epidemiology Biomarkers & Prevention. 2010;19:229–39.

5. Oldridge DA, Banerjee S, Setlur SR, Sboner A, Demichelis F. Optimizing copy number variation analysis using genome-wide short sequence oligonucleotide arrays. Nucleic Acids Research. 2010;38:3275–86.

6. Trapnell C, Pachter L, Salzberg SL. TopHat: discovering splice junctions with RNA-Seq. Bioinformatics. 2009;25:1105–11.

7. Li H, Handsaker B, Wysoker A, Fennell T, Ruan J, Homer N, et al. The Sequence Alignment/Map format and SAMtools. 2009;2078–9.

8. Mayba O, Gilbert HN, Liu J, Haverty PM, Jhunjhunwala S, Jiang Z, et al. MBASED: allele-specific expression detection in cancer tissues and cell lines. Genome Biology. 2014;15:405.

9. Habegger L, Sboner A, Gianoulis TA, Rozowsky J, Agarwal A, Snyder M, et al. RSEQtools: a modular framework to analyze RNA-Seq data using compact, anonymized data summaries. 2011;281–3.

| 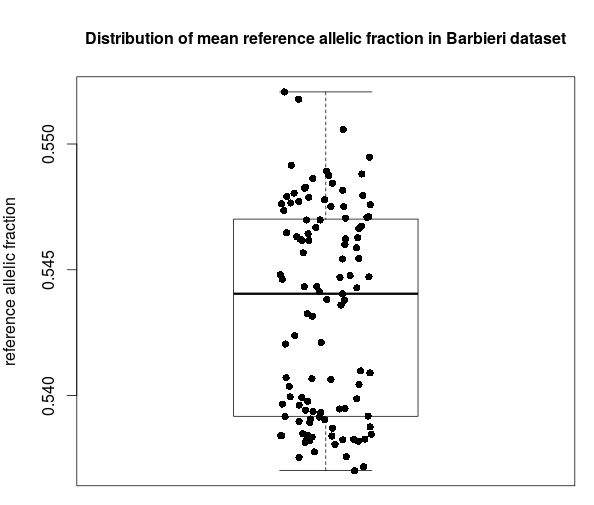 |
| --- |
| Figure S1: Distribution of mean reference allelic fraction from 111 normal WES samples of Barbieri et al. dataset. |
| 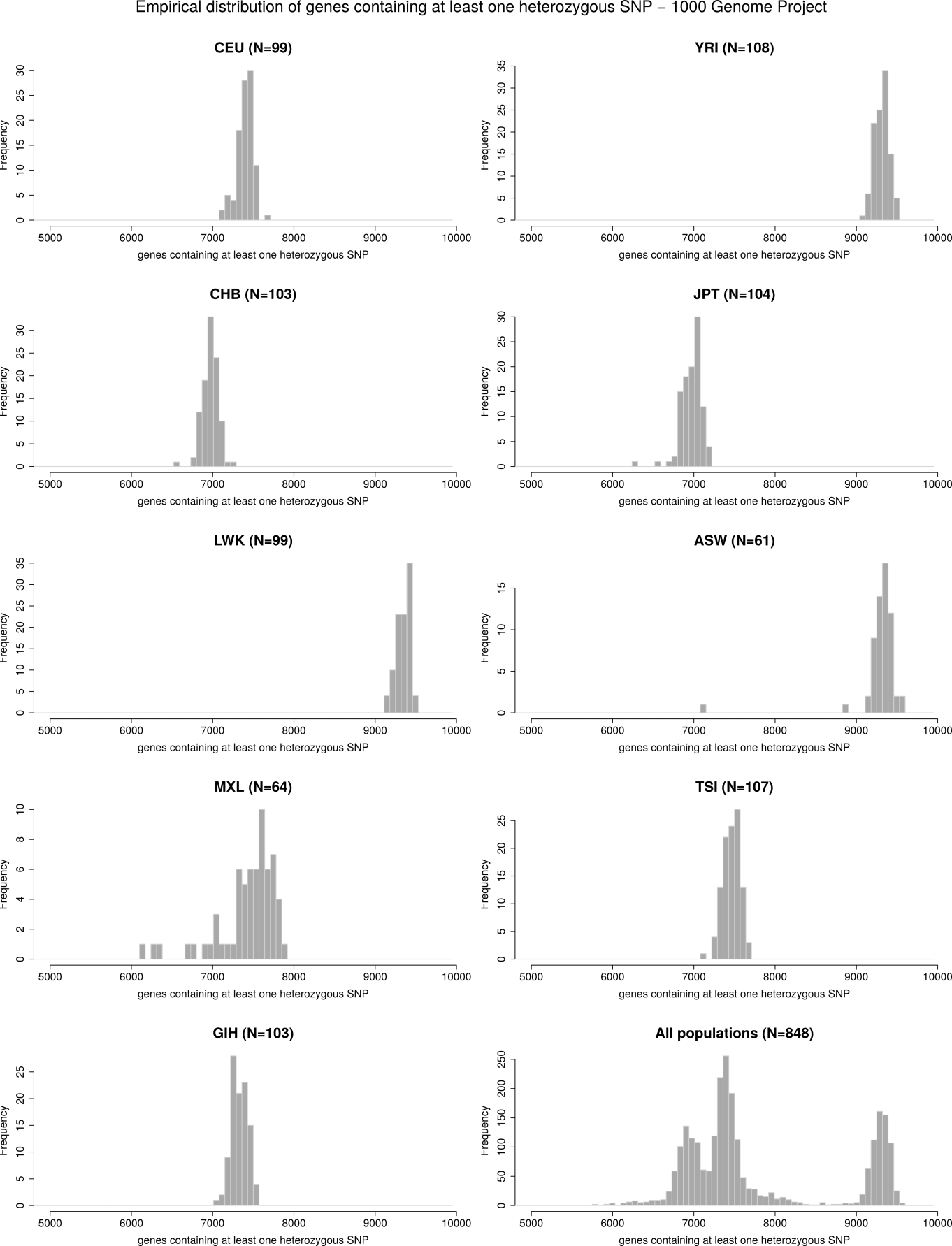 |
| Figure S2: Distribution of number of genes containing at least one heterozygous coding SNP across different populations from 1000 Genome Project data. Genotyping data of ~600000 coding SNPs for 848 samples across 9 populations were considered. For each sample the number of genes containing at least one heterozygous SNP is computed using the UCSC hg19 genes catalogue as reference. |
| 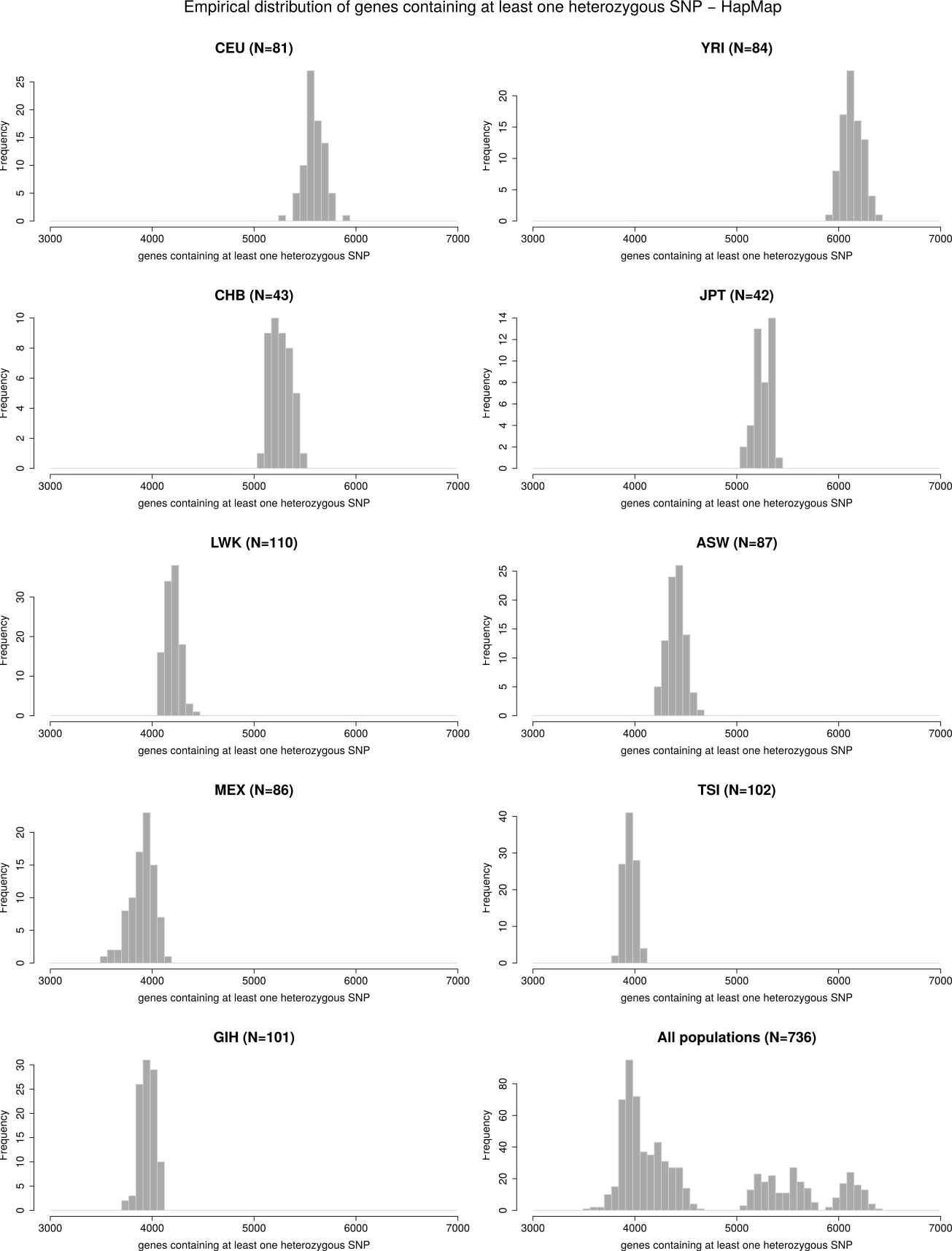 |
| Figure S3: Distribution of number of genes containing at least one heterozygous coding SNP across different populations from HapMap data. Genotyping data of ~200000 coding SNPs for 736 samples across 9 populations were considered. For each sample the number of genes containing at least one heterozygous SNP is computed using the UCSC hg19 genes catalogue as reference. |

| 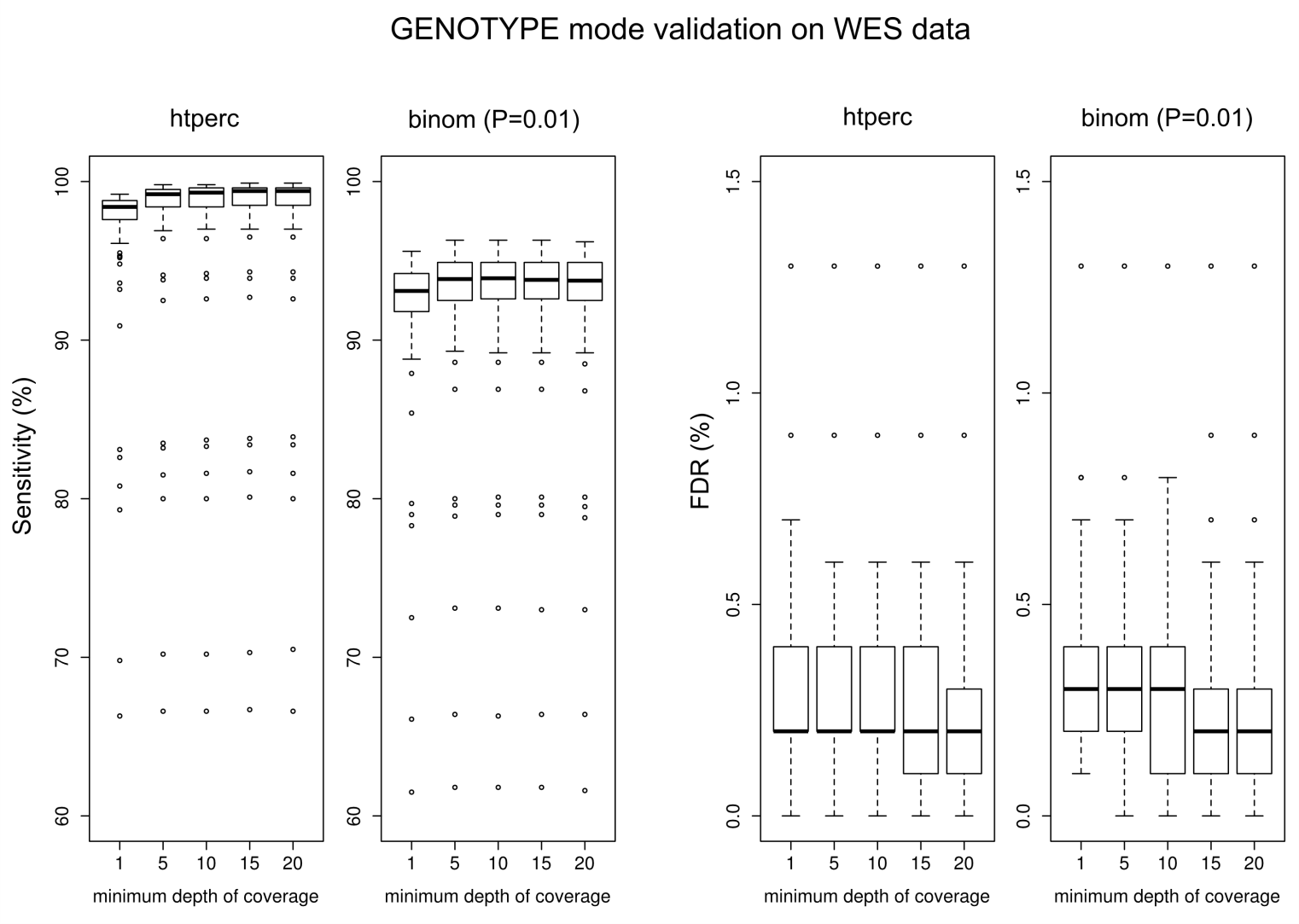 |
| --- |
| Figure S4: Sensitivity and FDR of GENOTYPE ASEQ method calculated on 90 WES sample from Barbieri et al. dataset. |
